# Pertussis re-emergence in the post-vaccination era

**DOI:** 10.1186/1471-2334-13-151

**Published:** 2013-03-26

**Authors:** Elena Chiappini, Alessia Stival, Luisa Galli, Maurizio de Martino

**Affiliations:** 1 Anna Meyer University Hospital, Department of Health Sciences, University of Florence, Florence, Italy

**Keywords:** Children, Pertussis, Vaccine

## Abstract

**Background:**

Resurgence of pertussis in the post-vaccination era has been reported in Western countries. A shift of cases from school-age children to adolescents, adults and children under 1 year of age has been described in the last decade, and mortality rates in infants are still sustained. We aimed to review and discuss the possible vaccination strategies which can be adopted in order to improve the pertussis control, by searches of Pubmed, and websites of US and European Centers for Disease Control and Prevention, between 1st January 2002, and 1st March 2013.

**Discussion:**

The following vaccination strategies have been retrieved and analysed: the cocooning strategy, the immunization of pregnant women and newborns, vaccination programs for preschool children, adolescents, adults and health-care workers. Cost-effectiveness studies provide some contrasting data, mainly supporting both maternal vaccination and cocooning. Adolescent and/or adult vaccination seems to be cost-effective, however data from observational studies suggest that this vaccination strategy, used alone, leads to a reduced pertussis burden globally, but does not affect the disease incidence in infants. Moreover, substantial logistical and economic difficulties have to be overcome to vaccinate the largest number of individuals.

**Summary:**

The simultaneous use of more than one strategy, including cocooning strategy plus vaccination of adolescents and adults, seems to be the most reasonable preventive measure. The development of new highly immunogenic and efficacious pertussis vaccines continues to be a primary objective for the control of pertussis.

## Background

Pertussis is still a major public health concern in Western countries where, despite high vaccination coverage, yearly incidence continues to increase and mortality in children under 6 months of age reaches 0.2% [1]. This trend has been reported in Canada, the United States and Australia since the 1980s and in Europe some years later [2]. Large outbreaks recently occurred in the United States, reporting impressive figures. As an example, during the 2010 Californian epidemic, over 9,000 cases have been recorded, for a rate of 23.4 per 100,000, the highest number in 60 years [3-5]. Similarly, in the UK in 2012 the highest mortality rate was registered since 1982, with 10 deaths, all occurred in infants under 12 months old [6]. In Europe, 27 countries currently provide national surveillance data for pertussis under the EUVAC-NET (European surveillance network for selected vaccine-preventable diseases): 17,596 confirmed cases were reported in 2009, corresponding to an incidence of 4.9 per 100,000 [7]. Data were heterogeneous among countries, ranging from 0.02 to 115 per 100,000. Pertussis rates were higher in Northern European countries, probably because some of them, including Sweden, Norway and Germany, achieved a high immunization coverage and introduced a booster dose after a primary immunization only recently. However, different rates may have been influenced not only by differences in vaccination policies, but also by differences in reporting procedures and surveillance systems, case definitions, and laboratory methods [2,7,8].

**Figure 1:**
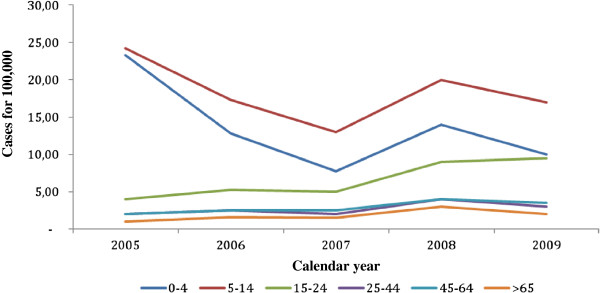
**Age-specific incidence distribution of pertussis cases in European countries, 2005–2009 (from **[13]**-**[17]**, modified).**

Possible reasons for the re-emergence of pertussis include the increased awareness of the disease, the development of new clinical definitions, and the spread use of polymerase chain reaction assays for laboratory confirmation, improving the diagnostic ability even in cases with atypical presentation [3,4,7,9]. Genetic changes in circulating strains of *Bordetella pertussis*, occurring under selective vaccination pressure, should also be considered [4,10]. Finally, protection from pertussis is not life-long, but restricted to a period of 5–8 years, after natural infection, as well as after vaccination [11]. This waning of immunity explains the shift of the incidence peak from school-age to adolescents/adults, and the spread from these subjects to infants and young children, still unvaccinated or not-fully vaccinated (Figure 1) [4,7,8,12-17]. Children under 6 months of age have a 20-fold higher rate of infection than the total population and ≥ 90% of pertussis deaths occur in this age class [18]. Aim of the present study is to review and discuss the possible vaccination strategies which can be adopted in Western countries in order to improve the pertussis control.

## Discussion

### Literature search

Data for this review were retrieved by searches of Pubmed, references from relevant articles and open-access websites of US Centers for Disease Control and Prevention (CDC) and European Centre for Disease Prevention and Control (ECDC). In order to verify the completeness of the PubMed database, we also performed the same key word searches with other databases (Web of Science, Embase, Pascal), but the results were virtually overlapping with regard to the subjects of interest, or supplied supplemental articles out of the scope of this review. The search was limited to English-language publications involving humans. The search has been performed in order to identify articles published between 1st Janury, 2002 and 1st March, 2013. In particular the search strategy used in the PubMed database was the following: “pertussis [Title] AND vaccine [Title]) AND (schedule [Title] OR strategy [Title] OR booster [Title] OR (cost [Title] AND effectives [Title]) OR efficacy [Title] OR pregnancy [Title] OR pregnant [Title] OR infants [Title] OR newborn [Title] OR adolescents [Title] OR (health-care [Title] AND worker [Title])) AND (hasabstract [text] AND “2003/02/16” [PDat] : “2013/02/12” [PDat] AND “humans” [MeSH Terms] AND English [lang])”. This search resulted in 132 articles which were reduced to 94 on the basis of titles and abstracts.

### Types of pertussis vaccines currently available in Western countries

In developed countries whole cell pertussis vaccines (wP) are not used anymore, due to the high rates of reported adverse events. In the 1970s and 1980s acellular pertussis (aP) vaccines were demonstrated to be effective, but less reactogenic than wP vaccines. As a consequence aP are now adopted in Western countries [19]. No preparation containing pertussis antigens alone is licensed in the United States or Europe to date [20]. Several pertussis vaccines are available combined with diphtheria and tetanus toxoids plus, eventually hepatitis B virus and/ or *Haemophilus influenza* type B and/or poliovirus antigens (i.e. Infarix, InfarixHepB, Infarix-hexa, Infarix-penta, Tetravac, Pentavac, Triacelluvax, Daptacel, Pentacel). They may include three antigens from purified *Bartonella pertussis* bacteria: pertussis toxin (PT), filamentous hemagglutinin (FHA) and pertactin (PRN) (i.e.: Infarix, Triacelluvax), or may be five-component vaccines additionally containing fimbrial antigen 2 (Fim2) and fimbrial antigen 3 (Fim3) (i.e. Daptacel, Pentacel) [21,22]. Currently, vaccines for the use in older subjects are also available (i.e. Boostrix, Adacel) containing reduced quantities (10-50%) of all antigens [20] to decrease the risk of injection site reactions occurring more frequently after the fifth dose of DTaP [23]. As an example, Boostrix is licensed for individuals from age 10 years onwards in the United States and from age 4 years onwards in Europe [24], while Adacel is approved in those aged 11–64 years in the United States and in children (aged ≥ 4 years), adolescents and adults in Europe [25].

### Efficacy and effectiveness data

Eight randomized controlled trials (RCTs) investigating the efficacy of pertussis vaccines have been retrieved (Table 1) [19,26,27]. Among these latter, 6 RCTs, overall including more than 46,000 participants, have been previously analysed in a Cochrane systematic review [19], demonstrating that the efficacy of multi-component (≥ three) aP vaccines is 84-85% in preventing typical whooping cough and 71-78% in preventing mild pertussis disease (Table 1) [9,19]. wP vaccines were found to be more efficacious than aP vaccines in some studies [28,29] but not in others [30]. Multi-component (three or five) aP vaccines showed higher efficacy than one- and two-component aP vaccines against both typical and mild pertussis disease, while data were insufficient to establish whether there was a clinically significant difference between three- and five-component aP vaccines [19]. Among the remaining two RCTs, not included in the Cochrane review, one study included about 83,000 children followed up for three years and the reported efficacy was 72.3% for the three component DTaP vaccine, 84.7% for the five component DTaP vaccine, and 89.1% for DTwP vaccine [26]. In another RCT, after a 2.5 year follow-up, efficacy of a three-component aP vaccine was 92% (95% CI: 32-99%) in 2,781 healthy subjects aged 15–65 years [27].

**Table 1:** Pertussis vaccine efficacy studies

**Study (year)**	**Country**	**Type of analysis**	**Included partecipans**	**Dose schedule**	**Type of vaccine**	**Efficacy of vaccine (95% CI)**
AHGSPV 1988	USA	Double bind parallel group RCT	Age 5 to 11 months	2 doses (entry + 8 to 12 week later)	aP: JNIH7	78% (57-88%)
					aP: JNIH6	78% (58-89%)
Trollfors 1995	Sweden	Double bind parallel group \ RCT	Full term, healthy infants	3 doses (3, 5, 12 months)	DTaP: Amvax	71% (63-78%)
Greco 1996	Italy	Double bind parallel group RCT	Age 6 to 12 weeks and weight >3rd percentile	3 doses (6 to 12, 13 to 20, and 21 to 28 weeks)	DTaP: SKB	84% (76-89%)
					DTaP: CB	84% (76-90%)
					DTwP: CON	36% (13-50%)
Gustafsson 1996	Sweden	Double bind parallel group RCT	Age 2 to 3 months	3 doses (2, 4, 6 months)	DTaP: SKB	59% (51-66%)
					DTaP: CON	85% (81-89%)
					DTwP: CON	48% (37-58%)
Simondon 1997	Senegal	Parallel group RCT	Age 2 months	3 doses (2, 4, 6 months)	DTaP: Pasteur-Merieux	85% (66-93%)
					DTwP: Pasteur-Merieux	96% (86-99%)
Olin 1997	Sweden	RCT	Age 2–3 months	3 doses (3, 5, 12 months or 2, 4, 6 months)	3-component DTaP	72%
					5-component	85%
					DTaP	
					DTwP	89%
PVSG 1998	Germany	Parallel group RCT	Age 2 to 4 months	4 doses (2 to 4, 4 to 6, 6 to 8, 12 to 14, and 15 to 18 months)	DTaP: Lederle/ Takeda	79% (72-85%)
					DTwP: Lederle	84% (77-89%)
Ward 2006	USA	Multicenter, double-blind RCT	Age 15 to 65 years	A single dose of a 3-component aP vaccine	aP (PT, FHA, PRN)	92% (32-99%)

Abbreviations.

RCT: randomised controlled trial.

aP: acellular pertussis vaccine.

DTaP: diphtheria-tetanus-acellular pertussis vaccine.

DTwP: diphtheria-tetanus-whole-cell pertussis vaccine.

SKB: SmithKline Beecham.

CB: Chiron-Biocine.

CON: Connaught.

Besides efficacy data reported in RCTs, a lot of information is available regarding vaccine effectiveness. In a US study, including more than 1,000 children, aged 6 months to 5 years, the estimated DTaP effectiveness was 83.6% for 1–2 doses, 95.0% for three doses and 97.7% for 4 or more doses [31]. In a cross-sectional study conducted in 272,000 Australian adolescents (12–19 years) with a three-component Tdap showed a vaccine effectiveness of 78.0% (95% CI: 60.7-87.6) [32].

Waning protection over years after aP vaccine has been reported, but data largely differ across studies. Laugauer and colleagues observed an effectiveness of 92% (95% CI: 84–9) for DTwP and 89% (95% CI: 79–94) for DTaP at 6 years follow up [33]. In an Italian unblinded prospective study including 9,554 children, effectiveness was 78-81% depending on the vaccine type during the first 6 years of life [22]. In a 1998–2009 UK study, vaccine effectiveness declined from 97.6% among infants 6–11 months of age to 83.7% among children 12–16 years of age (95% CI 69.5%–90.8%; p < 0.001) [34]. In another UK observational study, however, effectiveness declined to 52% in the fifth year after vaccination and to 46% in the seventh year after vaccination [35]. In a recent report from the 2010 California pertussis outbreak including about 170 paediatric cases, the reported vaccine effectiveness for a primary series and booster doses at 12–18 months and 4–6 years of age was 41% for children aged 2–7 years, but only 24% for children aged 8–12 years, suggesting waning immunity over time [36]. In interpreting such results it should be considered that in some circumstances small coverage variations could markedly change effectiveness observed. For example, in the Campbell’ s study, effectiveness for patients aged 10–16 years who received the DTwP vaccine under the accelerated schedule would increase from 82% to 90% if coverage increased by one per cent, from 97.7% to 98.7% [34].

### Pertussis vaccination schedules currently adopted in Western countries

Different vaccine strategies currently adopted in Western countries have been summarized in Table 2[7,37,38]. The primary immunization series usually consists of three consecutive doses during the first year of life, followed by a fourth dose in the second year of life and a fifth dose in preschool age [7]. Regarding booster schedules in adolescents, adults, including pregnant women and health care providers, recommendations vary considerably, as reported in Table 2.

**Table 2:** **Current pertussis vaccination schedules in Western countries (modified from**[7]**,**[37]**, and**[38]**)**

	**Age at primary series (months)**	**Childhood and adolescent boosters**	**Adult boosters**
Austria	3, 5 e 12	7-9 years and 13–15 years (only for those who previously received a Td booster)	Every 10 years
Belgium	2, 3 and 4	15 months, 5–7 years and 14–16 years	Cocoon, health-care workers, adults in contact with young children, day-care personnel
Finland	3, 5 and 12	4 years and 14–15 years	Adults (who did not receive any pertussis vaccination in the past 10 years), all health-care workers and cocoon
France	2, 3 and 4	16-18 months and 11–13 years	Cocoon, young adults (booster at 26–28 years)
Germany	2, 3 and 4	11-14 months, 5–6 years and 9–17 years	Every 10 years, cocoon
Italy	3, 5 and 11	5-6 years (and 11–15 years*)	..
Netherlands	2, 3 and 4	11 months and 4 years	Cocoon, pregnant women
Poland	2, 4 and 6	16-18 months and 6 years	..
Switzerland	2, 4 and 6	15-24 months and 4–7 years; (11–15 years catch-up)	Cocoon, young adults (booster at 25 years, 26–29 years catch-up, and to adults of any age in personal or professional contacts with infants ≤ 6 months
Canada	2, 4 and 6	16-18 months, 4–6 years and 14–16 years	Adults
United Kingdom	2, 3 and 4	3-5 years	Cocoon, pregnant women
United States	2, 4 and 6	15-18 months, 4–6 years and 11–12 years	Cocoon, pregnant women, health-care workers, adults (who did not receive any pertussis vaccination in the past 10 years)
Australia	2, 4 and 6	4 years and 11–12 years	Adults planning a pregnancy, cocoon (including grand-parent), adults who work with young children (child-care workers and health-care workers)

*Only in some regions.

### Possible implementation strategies to be adopted in Western countries

#### Vaccination of women during pregnancy

**Figure 2:**
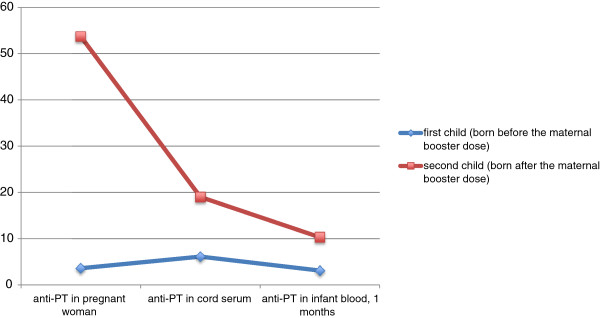
**Geometric mean titers (GMT) for anti-pertussis toxin (PT) antibodies in women and children before and after a maternal booster dose (from **[44]**, modified).**

Several countries, including US and UK, currently recommend Tdpa administration in pregnant women (Table 2) [39]. Safety data on Tdap vaccination in pregnant women are limited, however existing Tdap data from the CDC, US Food and Drug Administration and the pharmaceutical pregnancy registries do not indicate a safety signal [40]. A recent US survey based on Vaccine Adverse Event Reporting System (VAERS) data, including 2 reports of Tdap administered to pregnant women, did not identify any concerning patterns in maternal, infant, or fetal outcomes [41]. Previous studies have shown that the levels of pertussis antibodies are so low in unimmunized or incompletely immunized mothers that they will be undetectable in their infants’ blood within 2 months of age [42]. Efficient transplacental antibody transfer and significantly higher titers have been found in 1-month-old infants, born after a maternal booster vaccination, compared with siblings born before the maternal booster (Figure 2) [43,44]. A recent report assessed paired maternal and umbilical cord sera collected from 52 women immunized with Tdap during pregnancy compared with 52 women who were not. The data indicated that newborns born to mothers who received Tdap during pregnancy had significantly higher antibody titers to diphtheria anti-toxin (p < 0.001), tetanus antitoxin (p = 0.004), PT (p < 0.001), FHA (p = .0002), PRN (p < 0.0001) and fimbriae type 2/3 (p < 0.001) when compared with newborns born to unimmunized mothers [40]. However, it is uncertain whether this increase in antibodies can be considered clinically protective because no serological correlate of protection is universally accepted for pertussis [40]. Healy *et al*. have recently stressed the importance of timing of maternal Tdap immunization [45]. Maternal vaccination is ineffective during early weeks of gestation because of the rapid decay of antibodies. It should be administered during the third trimester to have maternal pertussis antigen-specific IgG levels at their peak when placental transport is the most efficient as possible. This may protect the infant during the immediate postpartum period when he is more vulnerable. Moreover, immunization should be repeated in each subsequent pregnancy [40,45].

Concern about the potential interference of maternal pertussis antibodies with infant immune response to primary DTaP vaccination has been raised [44]. It seems that the presence of circulating maternal antibodies can inhibit active pertussis-specific antibody production in the child. This blunting might reduce protection after first months of life [18,39,46,47]. A 1995 study by Englund and colleagues included 2,342 infants, who were randomized to receive DTaP or DTP vaccines at 2, 4, and 6 months of age. After DTP but not DTaP, higher levels of preexisting antibody were associated with substantial (28% to 56%) reductions in the subsequent antibody response to pertussis toxin (PT). This finding suggests that the use of aP vaccines in adults, which could confer higher levels of antibody in women before pregnancy, would be unlikely to adversely affect pertussis antibody responses after DTaP among infants born to mothers with high antibody levels [48]. Currently, two clinical trials are underway in Canada and the United States to measure the response to routine active immunization in the infants whose mothers received Tdap vaccine during the third trimester of pregnancy [49,50].

#### Immunization of newborns

Immunization of newborns is another possible strategy that has been investigated in some recent studies with the rationale to provide protection in first months of life when infants are more vulnerable. Halasa *et al*. analysed the results of neonatal vaccination with DTaP vaccine in 50 infants between 2 to 14 days of age. The administration of an additional dose at birth was safe and well tolerated, but was associated with lower geometric mean antibody concentration for toxin and pertactin at 6, 7, and 18 months, for fimbrae at 6, 7, 17, and 18 months, and for FHA at 18 months and lower geometric mean antibody concentrations for diphtheria at 7 months [51].

In contrast, two other studies administering only aP at birth, without diphtheria or tetanus toxoids, followed by the DTaP series at 2, 4 and 6 months, reported an enhanced immune response against pertussis antigens at 2 and 8 months of age but lower levels of antibodies against *Haemophilus infuenzae* type B and hepatitis B [52,53]. However, at least 96% of subjects achieved antibody concentrations associated with seroprotection after a booster dose of DTaP-HBV-IPV/Hib at 12 to 23 months [54]. An Australian study assessed the immunogenicity and reactogenicity of two doses of aP vaccine, one given at birth and the other one at 1 month. Data suggest that aP vaccine administered before 2 months of age induces significantly higher pertussis antibody titers by 2 months of age without interference with responses to routine active immunization [55].

The immaturity of the neonatal immune system and the impact of passively transferred maternal antibodies could explain the poor immune response in infants vaccinated at birth [43]. In infants DTaP vaccination may trigger CD4^+^ T-lymphocytes functionally and phenotypically dissimilar from those of older children and adults [56]. Given these controversial reports, currently, immunization with DTaP or aP vaccines is not recommended in newborns. Additional clinical trials are needed in this regard [18].

#### Cocooning strategy and postpartum mothers’ immunization

Cocooning strategy consists of providing indirect protection to infants who are too young to be immunized or protected by vaccine through immunization of their parents and other family members, caregivers and close contacts [7,18]. Household members, particularly mothers, are the source of transmission of pertussis to infants in up to 75% of cases [57]. However, casual community contacts have been estimated to account for up to 34% of cases [58]. There are very few empirical studies examining the impact of cocooning strategy. In a US cross-sectional study including more than 500 infants, immunizing only postpartum mothers with Tdap did not reduce pertussis cases in infants ≤6 months of age [59,60] suggesting that efforts should be directed at immunizing all household and key contacts of newborns, not just mothers. It should also be considered that a maximum response to Tdap is not achieved until 14 days after vaccination and in this gap newborns are at risk of infection [61]. Despite the paucity of data, economic and logistical difficulties, efforts to promote and effectively implement cocooning in Western countries is continuing [18]. In 2006 the Advisory Committee on Immunization Practices (ACIP) recommended routine administration of Tdap to all unvaccinated postpartum mothers and in 2011 recommendations were extended including pregnant women and all people who have or anticipate having close contact with an infant aged <12 months if they have not previously received it [39]. During the 2010 Californian epidemic the cocoon strategy was adopted together with other strategies such as adolescent and adult boosters, resulting in an incidence decline from 23.4 to 2 cases/100,000 in one year [62]. Given the majority of studies currently available are from the United States, these results may not necessarily be applicable to other settings. It is crucial that the countries where cocooning has been implemented investigate its impact on pertussis incidence to clarify its cost-effectiveness [63].

#### Vaccination of preschool children and adolescents

A preschool booster is usually included in the vaccination schedules of many countries (Table 2). It contributes to increase herd immunity and to reduce transmission to susceptible subjects [7]. A case–control study was conducted in California from 2006 to 2011, involving children who were vaccinated with all the five DTaP doses. The aim was to establish the risk of pertussis in relation to the time since the fifth DTaP dose. In this study protection against pertussis waned during the 5 years after the fifth dose of vaccine and the risk of disease increased by 42% each year [64]. Since the highest incidence of the disease is currently reported among adolescents, a universal booster vaccination in this age class has been proposed. In one RCT efficacy of Tdap in adolescents/adults was 92% [27], but the reported effectiveness is lower. In 499 adolescents, during a pertussis outbreak in a US school, vaccine effectiveness was only 65.5% (95% CI: 35.8-91.3%) [65]. In Australia, Tdap was administered from 2004 for 272,000 adolescents (aged 12–19 years) during a mass vaccination program. Vaccine effectiveness was evaluated by the screening method and it was 78.0%. The Australian experience supported the positive impact of a large use of Tdap to rapidly control pertussis in adolescents and suggested that a school-based catch-up program followed by immunization of school entrants might be the optimum strategy for the implementation of adolescent coverage [32,66].

Since 2005, the ACIP has expanded the routine adolescent vaccination schedule with the administration of one Tdap dose. Therefore, from 2006 to 2009, Tdap coverage among US adolescents increased from 10.8% to 55.6% and then it reached 78.2% in 2011, but it still remains below target levels [67]. Currently, the American Academy of Pediatrics recommends that adolescents 11 to 18 years of age should receive a single booster dose of Tdap instead of tetanus and diphtheria toxoids (Td) vaccine (the preferred age is 11 to 12 years). Those who have received Td but not Tdap are encouraged to receive a single dose of Tdap with a suggested interval of at least 5 years between Td and Tdap to reduce the risk of local and systemic reactions. The primary goal is to protect immunized adolescents against pertussis. A secondary object is to reduce the reservoir of pertussis within the population and prevent indirectly pertussis cases in infants and young children, who have the highest risk of complications [20]. Although a decreasing trend of pertussis cases was observed in adolescents after the introduction of Tdap vaccination, the average incidence among infants younger than 1 year did not change [68]. In Europe only few countries have introduced booster doses for adolescents (eg, Austria, Belgium, Finland, France, Germany and some Italian regions) (Table 1) [7,69]. Data on effectiveness of adolescent boosters in these countries are lacking [70]. Furthermore, at the Global Pertussis Initiative meeting in Paris 2010, members stressed the need of efficacious surveillance systems to evaluate the impact of dTap booster on the disease incidence in adolescents [66,69].

#### Vaccination programs for adults

Reports from Europe and the United States highlight the growing burden of pertussis in adult population [7]. Since 2005, the ACIP has recommended a Tdap vaccine booster dose for those adults aged 19 through 64 years who have not yet received a dose or have received their last dose of Td ≥10 years earlier and they have not previously received Tdap [71,72]. Despite these recommendations, Tdap coverage remained low in the US adults. In 2008 only 5.9% of adults received a dose of Tdap and coverage among adults with infant contact was estimated to be 5% [9].

In October 2010, ACIP recommended that unimmunized adults aged ≥ 65 years shall be vaccinated with Tdap if in close contact with an infant, and that other adults aged ≥ 65 years may receive Tdap. In February 2012, ACIP recommended Tdap vaccination for all adults aged ≥ 65 years [72]. Universal adult vaccination is an important strategy to build up herd immunity and eradicate pertussis infection [73]. In a randomized, multicenter, double-blind, controlled trial called APERT (Acellular Pertussis Vaccine Trial), 2781 healthy subjects between the ages of 15 and 65 years were recruited and received a single dose of either an acellular pertussis vaccine or a hepatitis A vaccine (control). It was estimated that a single dose if Tdap gave a protective efficacy of 92% among adolescents and adults [27]. A cost-benefit analysis showed that decennial adult booster vaccination, although more expensive than adolescent boosting, could prevent 0.9-4.7 million adult cases of pertussis and save $1.3-6.4 billion in the US every 10 years [74].

In the past decade, the confusing recommendations about Tdap immunization and the lack of precise guidelines resulted in underuse of the vaccine [75]. A universal decennial Tdap booster program should be implemented starting in preadolescents and continuing throughout adulthood, including persons aged ≥65 years. The Consensus on Pertussis Booster Vaccination in Europe group proposes the administration of a single dose of Tdap instead of dT in adults aged 18 years or older who have received the preceding dT dose for more than 10 years. Epidemiological studies are needed to define the appropriate interval of time between the two boosters [7]. Until now only a French study has assessed this problem. It concluted that Tdap-IPV (inactivated poliovirus) may be administered to adults as little as one month after Td-IPV without exacerbating post-vaccination side-effects [76]. Although Tdap booster dose 10 years later the initial booster has been proven to be equally immunogenic and well tolerated [77], there is currently paucity of data regarding the incidence of local reactions after repeated immunizations in adults. Some authors advocated the production of a monovalent acellular vaccine without diphtheria and tetanus toxoids which could allow more frequent boosters in adults [78].

#### Immunization of health-care workers

Health-care workers are at a higher risk of infection than the general population and, in turn, may be a substantial source for susceptible individuals. As an example, in one single-center study, 17 pertussis cases were identified, which exposed 355 unprotected health-care workers [79]. Pertussis among health-care personnel has been reported to be 1.7 times higher than among the general population [72]. In 2006, the Health-care Infection Control Practises Advisory Committee supported the recommendations of ACIP for the use of Tdap in health-care providers. It was proposed that health-care personnel who work in hospital or ambulatory care settings and have direct contact with patients should receive a single dose of Tdap as soon as feasible if they have not previously received Tdap; an interval as short as 2 years from the last dose of Td was recommended. The aim was to protect health-care workers against pertussis and to reduce transmission to patients. Priority had to be given to those ones in frequent contact with pregnant women, infants, children or immune compromised patients [71]. However, in 2008 Tdap vaccination coverage was only 15.9% among the United States health-care workers [9].

In some European countries (eg, France and Belgium), Tdap boosters for health-care workers are recommended (Table 2) [7]. In 2007, the French National Institute for Health Surveillance analysed data about nosocomial infections and community clusters of pertussis in France, reported between 2000 and 2005. Almost half of the 67 reports analysed were coming from hospitals and health-care workers were usually the first to be affected [80,81]. Studies are needed to evaluate the effectiveness of Tdap immunization in preventing pertussis in health-care providers and in their contacts, and to establish the duration of protection [71].

### Cost–effectiveness, cost-utility and economic impact model studies

In a recent review Millier and colleagues identified 13 cost-effectiveness, cost-utility and economic impact models regarding the impact of adolescent booster, one-time adult booster, adult decennial boosters and/or cocoon strategy. Adolescent booster was found to be a cost-effective strategy compared with no booster vaccination in all the nine considered studies [82]. As an example, Purdy *et al*. showed that immunizing adolescents aged 10–19 years would be the most economical strategy since it would prevent 0.7-1.8 million pertussis cases and save $0.6-1.6 billion over a decade in US [74]. However, in another recent review including 16 studies using a dynamic model, adolescent vaccination was found to be cost effective, but not highly effective in protecting infants too young to be vaccinated [83]. Similarly in another recent study, using an age-structured compartmental deterministic model, a single Tdap dose at age 11 years significantly would reduce the incidence of the disease within this age group, but would have a very low impact in infants [84].

The conclusions concerning adult vaccination, alone or in combination with adolescent vaccination, are also contrasting. A US cost-benefit analysis concluded that, although more expensive than adolescent boosting, decennial adult booster vaccination could prevent 0.9-4.7 million adult cases of disease and save $1.3-6.4 billion every 10 years [74]. In a recent study from Netherlands, combining an adolescent booster dose at the age of 10 years (most cost-effective age for a single adolescent booster dose) with an adult (18–30 years) booster dose resulted in favourable incremental cost-effectiveness ratios (ICERs) in terms of quality-adjusted life years (QALYs) (<€10,000/QALY) and the every 10 year booster dose resulted in an ICER of €16,900 per QALY [85]. On the other hand, in a German study adult vaccination would be cost-saving only if the incidence were higher than 200 per 100,000 and Lee *et al.* estimated that only 1.4% of cases would be prevented and adult booster strategy should not be adopted [82,86].

Available studies generally suggested the cost-effectiveness of the cocoon strategy, despite some conflicting results. In a Netherland study cocooning obtained by immunization of both parents was the most expensive intervention to implement but also the most effective. The base-case analysis suggested a reduction in the overall number of pertussis cases in infants by 26% [87]. Coudeville *et al.* in an economic evaluation including the dynamic population effects, concluded that the cocoon strategy complemented by a single booster dose was the most cost-effective one, and was associated with a 80% reduction of pertussis costs [88]. Differently, in a study by Lee *et al*., postpartum vaccination was found to be more costly than adolescent vaccination and would provide fewer health benefits [82]. In a recent Canadian study Skowronsky *et al*. suggest that parent immunization is inefficient and expensive in areas where disease incidence is low. In this setting the number needed to vaccinate should be at least 1 million to prevent 1 infant death, approximately 100,000 to prevent 1 infant ICU admission and more than 10,000 to prevent 1 infant hospitalization [59,89]. In Australia, Scuffham *et al*. reported an ICER of AUS$787,504 per DALY (disability-adjusted life-year) avoided versus no current schedule [90]. Parental vaccination would reduce pertussis cases, deaths and DALYs by 38.6%, 38.2%, and 38.3%, respectively. Nevertheless, it was not cost-effective, and dominated by the at-birth vaccination strategy [82].

Regarding the vaccination of pregnant women, some data are available supporting this strategy as cost-effective [39,61]. In a recent US study immunization during pregnancy was found to prevent a greater number of infant cases and deaths than postpartum one [91]. In a Netherland study the cost-effectiveness of cocooning and maternal vaccination were estimated to be similar, with ICERs of €4,600/QALY and €3,500/QALY, respectively [87]. It should be considered that these studies may be affected by abstract assumptions about unreported cases, real incidence, other epidemiological data, costs associated with mild disease and herd immunity effects [82].

### New possible vaccines

Currently available vaccines have clearly major limits and new vaccines are under developing in order to better control this disease. New vaccines could include additional protective antigens. Possible candidates include the adenylate cyclase toxin, the autotransporte BrkA, and an antigen induced by iron starvation, named IRP1-3 [21]. Another field of research is directed to develop a vaccine promoting the skewing of a predominant Th1 and Th17 immune response, which is the most effective [75]. Garlapati *et al*. studied a novel microparticle based vaccine formulation consisting of pertussis toxoid (PTd), polyphosphazene (PCEP), CpG ODN 10101 and synthetic cationic innate defence regulator peptide 1002 (IDR) against *Bordetella pertussis* in mice. Even if protection against pertussis is mediated by both humoral and cell-mediated immunity, several studies demonstrated that the Th1 and Th17 cell-mediated immune responses to initial doses of pertussis vaccines correlate better with long-term immunity than antibody levels. Investigators concluded that immunization with PTd encapsulated into microparticles and adjuvanted with CpG ODN and IDR induced a strong shift towards Th1/Th17 responses, with long-term immunity [75,92].

Another future objective is the development of a more immunogenic and efficacious vaccine using different immunization route and/or live attenuated vaccines [75]. Some researchers obtained a highly attenuated *Bordetella pertussis* strain that was able to colonize the mouse respiratory tract and to provide full protection after a single intranasal administration. These results provided hope for the development of novel vaccination strategies that could be used in the very young children, even at birth [93]. The intranasal route mimics the natural route of infection, stimulating mucosal immunity in addition to the systemic immune response. It could induce longer term protection than that offered by the currently marketed aP vaccines [94].

However it should be considered that he current DTaP vaccines are the basis of the infant immunization series and to replace them with new vaccines will require testing of all the other antigens. Thus, their use in the clinical practice could be difficult to be achieved in a short time period [21].

## Summary

Pertussis outbreaks continue to be reported in Western countries with high vaccination coverage. Despite the relevant efforts to protect all the groups at risk and interrupt the transmission of infection, the introduction of new strategies, including maternal vaccination, cocoon strategy, vaccinations in adolescents and adults have been suggested pursue this goal. All the strategies we have described focus on two of the several reasons for the on-going pertussis outbreaks, the inadequate levels of pertussis vaccination coverage in the population and the waning of vaccine-induced immunity in adolescents and adults over time. Other important causes must be considered: the loss of vaccine efficacy due to the emergence of new *Bordetella pertussis* strains and the possible skewing of pertussis immune responses in children due to use of the aP vaccine in early childhood. Cost-effectiveness studies provide some contrasting data, mainly supporting both maternal vaccination and cocooning. Adolescent and/or adult vaccination seems to be cost-effective, however data from observational studies suggest that this vaccination strategy, used alone, leads to a reduced pertussis burden, globally, but does not affect the disease incidence in infants. Moreover substantial logistical and economic difficulties have to be overcome to vaccinate the largest number of individuals. Policymakers should invest more resources in the education of public health providers and of the population about the benefits of vaccination. The development of new highly immunogenic and efficacious pertussis vaccines continues to be a primary objective for the control of pertussis.

## Competing interests

The authors declare that they have no competing interests.

## Authors’ contributions

EC and AS carried out the literature search and write the manuscript; LG and MdM reviewd and surpervised the all work. All authors read and approved the final manuscript.

## Pre-publication history

The pre-publication history for this paper can be accessed here:

http://www.biomedcentral.com/1471-2334/13/151/prepub
